# The Benefits of Telemedicine in Personalized Prevention of Cardiovascular Diseases (CVD): A Systematic Review

**DOI:** 10.3390/jpm11070658

**Published:** 2021-07-14

**Authors:** Gopi Battineni, Getu Gamo Sagaro, Nalini Chintalapudi, Francesco Amenta

**Affiliations:** 1 Telemedicine and Telepharmacy Centre, School of Medicinal and Health Products Sciences, University of Camerino, 62032 Camerino, Italy; getugamo.sagaro@unicam.it (G.G.S.); nalini.chintalapudi@unicam.it (N.C.); francesco.amenta@unicam.it (F.A.); 2 Research Department, International Radio Medical Centre (C.I.R.M.), 00144 Rome, Italy

**Keywords:** telemedicine, cardiovascular diseases, telecardiology, personal care, remote consultation

## Abstract

Introduction: Adverse effects on personalized care and outcomes of cardiovascular diseases (CVD) could occur if health systems do not work in an efficient manner. The pandemic caused by COVID-19 has opened new perspectives for the execution and advancement of cardiovascular tests through telemedicine platforms. Objective: This study aimed to analyze the usefulness of telemedical systems for providing personal care in the prevention of CVD. Methods: A systematic review analysis was conducted on the literature available from libraries such as PubMed (Medline), Scopus (Embase), and Cumulative Index to Nursing and Allied Health Literature (CINAHL). Data available in the last 10 years (2011–2020) were also examined by PRISMA guidelines. The selected studies were divided into two categories: (1) benefits of telemedicine in CVD prevention, and (2) recent progress in telemedical services for personalized care of CVD. Results: The literature search produced 587 documents, and 19 articles were considered in this review. Results highlighted that the timely delivery of preventive care for CVD which can be implemented virtually can benefit and modify morbidity and mortality. This could also reduce the pressure on hospitals by decreasing acute CVD occurrence among the general population. The use of these technologies can also help to reduce access to hospitals and other medical devices when not necessary. Conclusions: Telemedicine platforms can be used for regular checkups for CVD and contribute to preventing the occurrence of acute events and more in general the progression of CVD.

## 1. Introduction

In recent years, there has been a growing interest in the development of precision medicine approaches in the prevention and treatment of several pathologies with the purpose to offer to patients “the right treatment, to the right patient, at the right time”. In cardiovascular medicine, the potential of precision medicine applies to all stages of disease development and includes risk prediction, preventative measures, and targeted therapeutic approaches [1]. Telehealth and telemedicine enable precision medicine by two routes following data review by a doctor. These include a direct (online) discussion between a patient and doctor regarding a therapy (e.g., change dosing, prescription renewal), as well as prescriptions to the patient for them to undergo more precise testing and assessment. In both cases, the online session may add information or data to the patient history.

Telehealth and telemedicine, if properly used, may offer advantages for both patients and health professionals [2]. As per the World Health Organization (WHO), telemedicine can be defined as “the delivery of medical services, where distance is a basic factor, by all medical care experts utilizing information and communication technologies (ICT) for the exchanging of adequate information for the treatment, diagnosis and avoidance of illness and wounds, exploration and assessment, and for the proceeding with instruction of medical care suppliers, in the purpose of strengthening individuals health and their networks” [3]. Telemedicine includes the protected transmission of clinical information and data, by voice, text, images, or other forms required for the diagnosis, treatment, and patient follow up.

Telemedicine enables phone or video consultations to the benefit of patients and of healthcare professionals [4]. Doctors can conduct patient visits through a computer or smartphone. These types of services are providing a great opportunity to expand medical assistance, especially in the face of novel pandemics such as COVID-19 [5]. This pandemic forced the adoption of telemedical technologies, for avoiding interruption in patient care. Recent technological advancements have made telemedicine more feasible, even for people without good computer knowledge. It has been discussed whether telehealth services can fully replace face-to-face visits. In some cases, telemedicine is potentially equivalent to in-person meetings for cardiovascular diseases (CVDs) [6,7].

CVDs are the primary cause of a high number of deaths all over the world [8]. Furthermore, 80% of deaths caused by CVDs are associated only with strokes and heart attacks. People with a high risk of CVD have raised glucose levels and blood pressure, as well as obesity and overweight [9]. These can all be effectively estimated in primary care centers. Access to fundamental noncommunicable disease medications and basic technologies in healthcare guarantees doctor advice and treatment. In these situations, telehealth services can provide a great advantage to provide personalized care for patients with a high risk of CVD.

The telemedicine field applied to CVD is defined as telecardiology [10]. Personal medications or treatment can be suggested over video or phone conversations with the pill dispenser at hand. Family members or friends (from different locations) can participate in these visits and understand expert suggestions for improving the health literacy of the entire family. On the other hand, patients can remain at home with comfort, which allows welcoming talks with more involvement in decision making about their cardiac health, e.g., monitoring of blood pressure by taking acute readings with home devices. Frequent telemedicine visits can allow doctors to encourage patients to perform BP checkups. Studies have shown that self-observation improves both BP estimations and medicine adherence [11,12]. Moreover, hypertension is also an ongoing illness that stands at risk of CVDs and benefits largely from successive telehealth visits.

In this study, we conducted a systematic review analysis to explore the importance of telemedicine services in the provision of personalized care for CVDs. Moreover, we reviewed telecardiology advancements in the last decade and how the world is adopting telemedicine support in daily cardiologic practice.

## 2. Methods

### 2.1. Data Source and Search Strategies

Initially, we collected the available literature from high-quality medical libraries such as PubMed (MEDLINE) and the Cumulative Index to Nursing and Allied Health Literature (CINAHL). The Scopus (EMBASE) database was used for sorting out non-appearance articles from the two medical libraries. Medical Subject Headings (MeSH) were applied to identify key terms of MEDLINE. Under the ‘cardiovascular diseases and ‘telemedicine’ key terms, we were able to identify 63 and 11 subheadings, respectively, with the help of the MeSH list. The search string with the Boolean operator as ‘telemedicine’ AND ‘cardiovascular diseases’ for PubMed was used. For CINAHL, we included similar MeSH terms to those used in MEDLINE and, for Scopus (EMBASE), Boolean operators such as ‘telemedicine’ OR ‘telehealth’ OR ‘telecardiology’ AND ‘adoption’ OR ‘implementation’ AND ‘personalized care’ OR ‘personal medication’ OR ‘personal care’ AND ‘cardiovascular diseases OR ‘CVD’ OR ‘cardiology’ were applied.

### 2.2. Inclusion and Exclusion Criteria

Articles on telemedicine or telehealth in providing personal care for patients with a high risk of CVDs were considered for this research. Articles from peer-reviewed journals, featuring descriptive work in English language with full text, implementing cross-sectional and mixed methods (including both quantitative and qualitative), with objectives of telemedicine in cardiovascular care, were included. Exclusion criteria were (a) conference works, (b) abstract-only articles, and (c) articles published before 2011. Studies with irrelevant data on telemedicine and that did not mention telecardiology involvement were excluded from the study.

### 2.3. Quality Criteria

Once the literature search was done, the four authors independently assessed every article in two phases. In the first phase, duplicated or similar papers extracted from the three databases were removed by reading abstracts. This analysis was conducted by the conventional approach of reading the article title and abstract. After applying inclusion and exclusion criteria, the quality evolution of each selected item was done on the basis of the Newcastle–Ottawa Scale (NOS) that ranged from 0–9 [13]. The NOS defined each study in three ways as poor (0–4), moderate (5–6), and good (7–9). These scores were based on some filters such as study selection, comparability, and outcome. Various quality parameters such as demonstration, coherence, risk factors, and others were also considered. The quality scores of selected articles calculated using the above parameters were recorded in an excel sheet to calculate whether a selected study was suitable for final consideration or not.

## 3. Results

**Figure 1:**
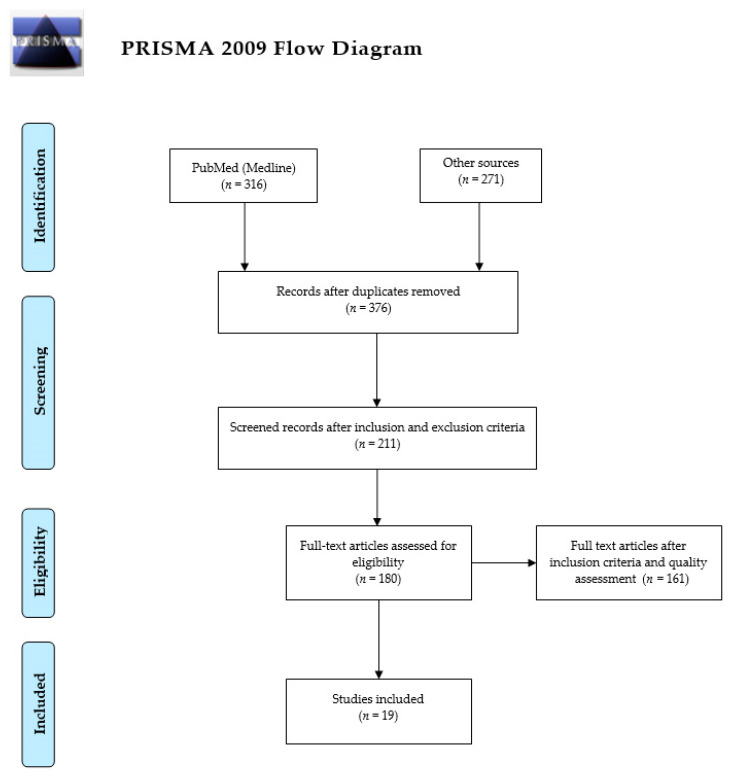
Records screening presentation by preferred reporting items for systematic reviews and meta-analyses (PRISMA) [14].

The literature search between 2011 and 2020 resulted in 587 documents; 376 documents were excluded because of duplication during the initial screening, and this resulted in 211 papers (Figure 1). In the subsequent phase, by applying exclusion criteria, 162 papers were excluded and 49 were left for quality assessment and further analysis. These documents were equally distributed to each author to perform multiple screenings with the request to provide the quality scores of each work anonymously. Items that were not strictly in line with review objectives and/or did not achieve good quality scores were not further considered. Ultimately, 19 studies were included for a qualitative synthesis analysis, and the results of these works were summarized in tabular form.

### 3.1. Summary of the Main Findings

All 19 studies considered suitable for our analysis were conducted in developed nations. This suggests that telemedicine technologies in CVD prevention are more ready to be adopted in developed areas. Considering cost constraints in telemedicine implementation, governments or private bodies should provide financial support. A high number of studies (15/19, 78.94%) were funded by external organizations including public (11/15, 73.3%) and private bodies (4/15, 26.7%) (Refer Table 1).

Most studies provided evidence on the efficiency and efficacy of telemedicine in the management of CVD risk factors such as obesity [15], vascular risk management [16], heartbeat connections [17], hypertension [18], and cardiac arrest [19]. Telemedicine also involved transmission of electrocardiograms [20], CVD intervention improvement [21], outpatient management [22], and cardiac rehabilitation [19]. Table 2 reports the studies involved in telemedicine for the personalized care of CVD patients. Main factors such as patient satisfaction [21,23,24], personal medication outcomes [15,22,25], self-management of CVDs [20,26], quality care [17,27,28,29], cost-effectiveness [30], and technical advancements [31,32,33] were also observed.

### 3.2. Study Characteristics

Papers reviewed in this work are listed in Table 3. The main results of the papers evaluated are detailed below.

#### 3.2.1. Telemedicine in CVD Treatment

Because of telemedicine platforms, medical services in CVD prevention are largely effective with regular checkups and counseling to strengthen prevention ideas. The advantages include less patient exposure and staff, use of the entire cardiovascular care team, improved opportunities for patient-provider information, and reduced specialist shortages.

##### Reduced Patient Exposure and Staff

It is estimated that telemedicine programs, largely involving patient monitoring by virtual communication and control of emergency room visits, reduce costs of healthcare by about 15% [30]. Currently, because of the global pandemic caused by COVID-19, patients with heart diseases or coronary failure are becoming a high-risk potential group for deaths. However, lockdown presents a solution for rapid viral spreading by minimizing regular hospital visits and direct medical assessment. In this scenario, telemedicine strategies can ensure constant care for patients with heart failure [34]. Virtual visits by remote monitoring features an audiovisual telemedicine communication system that can be helpful for communication between specialists and patients via online portals [34,35,36]. Telemedical systems can contribute to monitoring major symptoms, facilitating the provision of medical data to doctors.

##### Opportunities for Patient-Provider Information

Some medical hospitals launched telemedicine via 4G tablets to provide chronic care including CVD and chronic heart failure. Patients are equipped with Bluetooth biometric devices (e.g., pulse oximeter) to measure blood pressure, oxygen levels, weight, and temperature [37]. These devices automatically record and transmit data that can be used for improved home care monitoring. Remote monitoring of implanted devices is usually associated with the transmission of recorded data via an external transmitter to the provided database. The patient data are transmitted daily. This can improve the clinical outcomes [38], enhance patient satisfaction [39], and save patient data.

##### Internet-Based Physical Activities for Cardiovascular Care and Cardiac Rehabilitation

The recovery from acute coronary syndrome is largely associated with cardiovascular exercises. Provision of personal care to control risk is quite expensive. The Cardio Fit internet-based physical activity expert system was found to improve both objectively measured physical activity and quality of life in patients with coronary heart diseases (CHD). Physical activity levels were measured through a pedometer for all randomized subjects for 6 to 12 months [33]. These telemedicine systems are largely beneficial and easy to use for heart failure, and to the use of wireless devices can reduce doctor visits [33]. Some studies determined whether a program with wireless implantable hemodynamic monitoring systems (W-IHM) reduced rates of hospitalization in patients with heart failure over 6 months follow-up [40]. The primary endpoint was met, and the heart-related hospitalization rate was reduced by 37% in the treatment group. It has been proven that the internet can be effectively incorporated in successive management in measure of pulmonary artery pressure, leading to better care among control groups [16].

Telemedicine had a positive impact on the lifestyle habits of veterans [18]. In this respect, telemedicine application in cardiac rehabilitation could positively impact improper access to the emergency room by chronic patients such as patients with CVD, as well as improve their self-care behavior. Two studies emphasized the potential of telemedicine in controlling CVD risk factors through electrocardiograms [20,27]. Widmer et al. [19] investigated whether a digital health intervention (DHI) application during cardiac rehabilitation would reduce cardiovascular-related emergency visits and rehospitalization in patients after percutaneous coronary intervention (PCI) and acute coronary syndrome (ACS). Digital health-based cardiac rehabilitation programs significantly reduced cardiovascular-related emergency department visits and unnecessary rehospitalization [41]. To advance the steady advantage of eHealth interventions for patient behavior, information on major contextual narratives should help with improving adequacy, thereby stimulating further studies on communications in comparable intercessions. Some studies [23,26] presented these issues in a personalized medicine context and highlighted the necessity of e-health interventions in exploring the prevention of cardiovascular diseases among latent user groups.

##### Reduction in Specialist Shortages and Improvement of CVD Care Efficiency

Incorporating telemedicine technologies into remote healthcare systems can compensate for the scarcity of cardiologists, heart specialists, subspecialists, and cardiac surgeons. Telehealth systems in cardiac care can improve access and doctor workforce strategies [42]. This leads to better communication with patients, increases practice efficiency, enhances quality care, and addresses projected shortages in the medical workforce. An increase in the use of telemedicine is expected to impact healthcare access quality, care costs, and education. Telemedicine technologies applied to home care have the potential to impact to present cardiac care models and explore the communication among experts, leading to have high-quality, more efficient, and less expensive care [42,43]. These models can function as a platform to provide primary care and management of critical care patients in a sophisticated manner [39]. On the other hand, telemedicine technologies enable cardiac specialists working in remote areas with continuous medical education to improve their ability to care. This reduces the patient’s travel to the care center, along with family and friends.

#### 3.2.2. Telemedicine in CVD Prevention

Obesity is one of the risk factors for the prevalence of cardiovascular diseases. The effectiveness of telemedicine technologies in obesity intervention for patients diagnosed as overweight or obese has to be majorly studied. Phone-based intervention also improved all causes of hospitalization or mortality as the composed endpoint of patients. An RCT on 767 patients (mean age = 61 ± 15 years) was carried out to assess whether short message service (SMS) could help improve readmission and self-care behavior (medication compliance, weight monitoring, salt and water restriction, and exercise) of patients with chronic heart failure [44]. The study subjects were followed up until 180 days through telephone or clinical visits after discharge. It was reported that weight loss interventions through telemedicine can improve readmission, self-care behavior, and quality of life [45]. A significant weight loss was recorded by telemedicine compared to in-person coaching over 2 years.

Coronary heart failure is a major risk factor for CVD. A randomized controlled trial (RCT) among 223 patients (mean age = 56.4 ± 9.0) with CHD assigned into two groups, Cardio Fit (*n* = 115, mean age = 56.7 ± 9.0) and usual care (*n* = 108, mean age = 56.0 ± 9.0), was carried out to assess the effects of online coaching on physical activity intervention for CHD patients by comparing usual care over 1 year [33]. The Cardio Fit group received a personally tailored physical activity regimen and access to a secure website for activity planning and tracking. Online training was also given to the Cardio Fit group, and they were in email contact with an exercise specialist. In contrast, the usual care group received physical activity guidance from their cardiologist. The authors found that at the two time points (every 6 months), the Cardio Fit group took an average of 764 (12% absolute difference) more steps per day than the usual care group. Furthermore, it was shown that patients with heart failure can be discharged from the hospital to evaluate medication adherence via telephone [46].

Telemonitoring reduces the readmission of patients with heart failure and significantly decreases contact with specialized nurses on this matter [47]. Some studies highlighted that telemedicine and telemonitoring applications have the potential to transmit diagnostic images, data, or reports, virtually connecting the patients and health professional in the screening of CVD; telemedicine via telephone communication is particularly useful in the early detection of CVDs, thus reducing costs [27,28,29]. The study by Koehler et al. [48] randomized 1571 patients with heart failure to assess the effect of remote patient management intervention on unplanned cardiovascular admission and mortality. The authors randomly assigned the patients into two groups to receive remote patient management (*n* = 796) or usual care (*n* = 775). The percentage of days lost due to unplanned cardiovascular hospital admission or death from all causes was significantly reduced in patients allocated to remote patient management (ratio: 0.80; 95% CI: 0.60–1.00, *p*-value = 0.0460) when compared to usual care [17]. In this regard, supporting telemedicine interventions in patients with heart failure can reduce unplanned cardiovascular hospitalizations and all causes of mortality.

## 4. Discussion

The use of telemedicine as a support to cardiology was introduced more than 100 years ago in Australia with the use of the telegraph from Barrow Creek Telegraph Station. In 1905, Einthoven transmitted electrocardiographs using the telephone line. A further evolution of this experience dated in 1910 with tele-auscultation transmitted through the telephone lines. In 1935, the first tele-electrocardiographic service was established in Lviv in the Ukraine. In 1938, the International Radio Medical Center (C.I.R.M.) in Rome received by radiotelegraphy an electrocardiogram from a passenger ship. In the last 10 years of the last century and at the beginning of this century, telemedicine started to move from the experimental phase to large-scale experiences, and its practical applications related to cardiology are entering in the standards of medical practice.

In this systematic review, we attempted to understand the positive effects of telemedicine and telehealth technologies on controlling cardiovascular diseases. The assessment of articles incorporated in this study explained that each article obeyed the NOS quality checklist and scored at least 7 (NOS ≥ 7), indicating scientific rigorousness of the study selection. In addition to highlighting the telemedicine effectiveness in CVD management, this work described the recent advancements in the last decade. Through this work, the key aspects of telemedicine in terms of evaluation of its role in the perspective of CVD personal care were identified. We found 19 quality research studies from Australia, China, France, Italy, Netherlands, and the United States, among others.

During serious pandemics such as COVID-19, a huge decrease in hospitalization and expanded self-administration by patients embodies the financial discernment of telemedicine since it can decrease patient flow to hospitals. Telemedicine has provided a chance to see remote patients, which has helped doctors in checking and monitoring conditions that the patient or their close family can easily follow. Those patients infected with COVID-19 are commonly associated with hypertension, CVD, and diabetes. In this pandemic, there have been other situations raising concern for the personal safety of patients who are at high risk for CVD. In such cases, the adoption of cardiac rehabilitation techniques is mandatory, including cardiac rehab in a home, which can guarantee the continuity of these mandatory services [22].

The effect of technological advancements on financial issues in healthcare has exceptionally exposed the divergence between the innovation cost and the change (either increase or decrease) in medical resource utilization because of the clinical impact of this technology. For instance, current telemedical technologies can provide personal care to cardiac arrest patients, certainly amplifying the treatment costs in long-term care for stroke survivors. The difference in quality and cost gained has been highly influenced by the cost-effectiveness of telehealth technologies in the prevention of cardiovascular diseases. We identified two studies that mentioned the development of telemedicine tools, namely, the consumer navigation of electronic cardiovascular tools (CONNECT) [31] and a novel system based on mobile internet, cloud computing in management of hypertension, and experts in the monitoring of physical activity among heart disease patients [33]. These tools need to be enhanced to function for long-term care, and a time framework should be designed to estimate the quality of care of CVD risk patients.

However, most of the available literature associated with clinical trials and pilot projects provided short-term results. A few were associated with long-term usage of telemedicine in CVD care [36,38,39,40]. Some clinical trials reported the efficacy of telemedicine rather than effectiveness [16,18,19,21,29]. One study presented a community-based survey of CVD risk factors to evaluate the potentiality of telemedicine [27], and another study reported follow-up data of CVD patient health status or clinical outcomes who participated in telecardiology services [20]. Other reports on telemedicine involvement in CVD care produced a strong assumption regarding the possible benefit to health status monitoring although the availability of limited patient administrative data.

According to the literature, telemedicine has the benefit of saving time and providing personal care for the patient. Physicians have to consider the shortcomings of these technologies, whereas telemedicine could be expensive for hospitals or small healthcare centers to provide. Simultaneously, if the patient prefers direct visits to a doctor, telemedicine is not an ideal solution. Therefore, the doctor has to maintain bonding with a patient to make them comfortable with the new technologies. We also have to consider the fact that no service is perfect, and telemedicine is no exception. Studies have continuously proven that telemedicine can save money, time, and lives.

The present systematic review had some limitations. Search parameters such as full text in the English language, the limited time frame (2011–2020), and the use of three databases with fixed keywords reduced the quantity of the study materials. Therefore, some relevant or original studies from all developed countries might have been missed in this work. Second, as with most systematic reviews, there was a quality bias of selected articles. The involvement of a limited number of people (four authors) could have influenced the comprehensiveness of this work, whereas adopting quality criteria during paper screening could have hampered the conclusions.

## 5. Conclusions

The overall findings of this work highlight the importance of telemedicine and telehealth technologies in the management of personal care among CVD patients. Moreover, they provide evidence of the benefits to cardiovascular care when using telemedicine in developed nations. Many studies are available on telemedicine applications, but sophisticated qualified studies are still very few, and the generalization of most evaluation outcomes is rather limited. However, one positive finding is that the world is now understanding the importance of these technologies because of significant challenges introduced by COVID-19. There has at least been a start in some developing countries, with public bodies ready to improve the economic situation through the adoption of telemedicine. We strongly suggest future developments for the provision of medical services through telemedicine, along with necessary training for both patients and providers, thus resulting in better healthcare and enhanced patient satisfaction.

## Figures and Tables

**Table 1:** Studies with funding opportunities.

Funding Source	Frequency (%)
Public bodies	11 (57.89%)
Private bodies	4 (21.05%)
No funding	4 (21.05%)

**Table 2:** Studies reporting efficiency of telemedicine in CVD management.

Telemedicine in Personalized CVD	Frequency (%)
Obesity control	5 (26.31%)
Transmission of electrocardiograms	3 (15.78%)
Outpatient management	4 (21.05%)
Control of cardiac arrest	7 (36.84%)
Hypertension control	6 (31.57%)
Cardiac rehabilitation	4 (21.05%)

**Table 3:** Summary of findings for the role of telemedicine in cardiovascular disease/risk management.

*n*	Name of Author (S) and Year	Number of Participants	Type of Study	Advantage	Outcome
1	Walter V.D. et al., 2012 [30]	-	Cost analysis model	Measuring the impact of cost involvement in treatment, the incremental net benefit, and the quality-adjusted life years	15% decrease in emergency room visits, as well as changes in utilization costs among bypass (−17%), rehabilitation (−13%), catheterization (−59%), medication (−14%), and angioplasty (−59%).
2	Reid R.D. et al., 2012 [33]	223	RCT	Monitoring physical activity for patients with ACS	More effective in monitoring physical activity for patients with ACS than for patients who received usual care
3	Gallagher B.D. et al., 2017 [46]	40	RCT	Medication adherence in a patient with heart failure	Improved medication adherence
4	Abraham W.T. et al., 2011 [40]	550	RCT	Monitoring pulmonary artery hemodynamic data for a patient with heart failure to reduce hospitalization	Significant reduction in hospitalization for patients with heart failure.
5	Vernooij J.W.P. et al., 2012 [16]	330	RCT	Promoting self-management in reducing vascular risk factors	Effective in reducing vascular risk and risk factors for patients with vascular diseases
6	Chen et al., 2019 [44]	767	RCT	Reducing readmission and improving self-care of patients with chronic heart failure (CHF)	Improved self-care behavior and reduced days lost in readmission
7	Appel L.J. et al., 2011 [45]	415	RCT	Weight reduction intervention in obese participants.	Significant weight loss recorded by telemedicine compared to in-person coaching over 24 months.
8	Boyne J.J. et al., 2012 [47]	870	RCT	Monitoring to identify the early symptoms of patients with heart failure to reduce the readmission of rehospitalization	No significant result was found in reducing the rehospitalization
9	Marino M.M. et al., 2020 [49]	430	Cross-sectional	For screening and early detection programs in the prevention of CVD	Effective in screening, early detection, and cost reduction.
10	Dendale P. et al., 2012 [50]	160	RCT	Monitoring and following up patients with heart failure to reduce rehospitalization and mortality rate	Effective in collaboration with doctors to reduce death rate and several days lost due to hospitalization
11	Koehler F. et al., 2018 [48]	1571	RCT	Detecting early signs and symptoms of a patient with heart failure	Effective in detecting early signs and symptoms, as well as reducing days of unplanned readmission and causes of death
12	Benson G.A. et al., 2018 [17]	1028	Retrospective cohort study	For the intervention of main CV risk factors (dyslipidemia and hypertension)	Significant improvement in the prevention of dyslipidemia and hypertension (CV risk factors) among patients at high risk for developing CVD
13	Widmer R.J. et al., 2017 [19]	64	RCT	Cardiac rehabilitation and rehospitalization of patients after PCI for ACS	Significantly reduced CV-related emergency department visits and rehospitalization in patients after ACS
14	Heron N. et al., 2019 [41]	40	RCT	Improving home-based prevention program of patients with a transient ischemic attack	Improved secondary prevention after a transient ischemic attack
15	Bosworth H.B. et al., 2018 [18]	429	RCT	For self-management of patients with CV risk factors	Effective in the intervention of CV risk factors and self-management of patients with CVD
16	Joubert J. et al., 2014 [27]	91	Cross-sectional	Screening CV risk factorsat the community level	Effective in screening CV risk and sending data from a remote area
17	Brunetti N.D. et al., 2015 [20]	3213	Follow-up	Prehospital electrocardiogram screening and remote teleconsultations	Effective in detecting acute CVD via ECG
18	Wienert J. et al., 2019 [26]	310	RCT	For the intervention of health behavior related to CV risk	Effective in promoting a healthy lifestyle to reduce CV risk
19	Genevieve C. et al., 2020 [23]	934	RCT	Involving digital health with the integration of personal data of primary care	The study highlighted that EHR-integrated eHealth interventions have better potential to help the cognitive, affective, and behavioral characteristics of changing health behavior.

## Data Availability

Not applicable.
